# Cinnamein Inhibits the Induction of Nitric Oxide and Proinflammatory Cytokines in Macrophages, Microglia and Astrocytes

**DOI:** 10.33140/jcei.08.01.01

**Published:** 2023-01-31

**Authors:** Swarupa Pahan, Sumita Raha, Sridevi Dasarathi, Kalipada Pahan

**Affiliations:** 1 Division of Research and Development, Jesse Brown Veterans Affairs Medical Center, Chicago, USA;; 2 Department of Neurological Sciences, Rush University Medical Center, Chicago, USA

## Abstract

Chronic inflammation driven by proinflammatory cytokines (TNFα, IL-1β, IL-6, etc.), and nitric oxide (NO) plays an important role in the pathogenesis of several autoimmune, inflammatory as well as neurodegenerative disorders like rheumatoid arthritis, multiple sclerosis, Alzheimer’s disease, Parkinson’s disease, Huntington’s disease, etc. Therefore, identification of nontoxic anti-inflammatory drugs may be beneficial for these autoimmune, inflammatory and neurodegenerative disorders. Cinnamein, an ester derivative of cinnamic acid and benzyl alcohol, is used as a flavoring agent and for its antifungal and antibacterial properties. This study underlines the importance of cinnamein in inhibiting the induction of proinflammatory molecules in RAW 264.7 macrophages and primary mouse microglia and astrocytes. Stimulation of RAW 264.7 macrophages with lipopolysaccharide (LPS) and interferon γ (IFNγ) led to marked production of NO. However, cinnamein pretreatment significantly inhibited LPS- and IFNγ-induced production of NO in RAW 264.7 macrophages. Cinnamein also reduced the mRNA expression of inducible nitric oxide synthase (iNOS) and TNFα in RAW cells. Accordingly, LPS and viral double-stranded RNA mimic polyinosinic: polycytidylic acid (polyIC) stimulated the production of TNFα, IL-1β and IL-6 in primary mouse microglia, which was inhibited by cinnamein pretreatment. Similarly, cinnamein also inhibited polyIC-induced production of TNFα and IL-6 in primary mouse astrocytes. These results suggest that cinnamein may be used to control inflammation in different autoimmune, inflammatory and neurodegenerative disorders.

## Introduction

Inflammation is a natural response of the immune system after an injury or infection to combat the insult and heal. While short-timed inflammation is beneficial for recovery after the insult or injury, prolonged inflammation (chronic) is harmful as it is capable of initiating the onset and/or aggravating the progression of several autoimmune, inflammatory and neurodegenerative disorders like rheumatoid arthritis (RA), multiple sclerosis (MS), Alzheimer’s disease (AD), Parkinson’s disease (PD), Huntington’s disease (HD), etc. Although several anti-inflammatory drugs are available in the market to ease pain, swelling, fever, and other symptoms of inflammation, most of the anti-inflammatory drugs have side effects. For example, prolonged use of Advil^®^ (Ibuprofen) and Tylenol^®^ (Acetaminophen) causes ulcerations of stomach and liver, respectively, leading to bleeding [1–3]. Antimalarial drug hydroxychloroquine often prescribed for RA has significant side effect [4]. Use of prednisolone and corticosteroids leads to compromised immune system [5]. Chemotherapy drugs like Methotrexate^®^ and Cyclophosphoride are also prescribed for inflammation, but at a lower dose for less side effects. Therefore, there is an absolute need for a non-toxic, safe, economic, and easily administrable anti-inflammatory medication that can be beneficial to millions suffering from different inflammatory diseases.

Cinnamein, chemically known as benzyl cinnamate, is naturally available in Balsam of Peru and Tolu Balsam [6, 7]. It is non-toxic and white to pale yellow crystalline solid with a sweet odor. Cinnamein is used as a flavoring agent and pharmaceutically utilized for its antibacterial and antifungal properties in tropical formulation, Sudocrem, an over-the-counter medicated cream primarily for the treatment of nappy rash [8]. This study was undertaken to examine whether cinnamein could be repurposed for controlling inflammation. Here, we present evidence for the first time that cinnamein markedly inhibited the release of different proinflammatory molecules in mouse RAW 264.7 macrophages and primary microglia and astrocytes. These results raise the possibility that this naturally-available non-toxic compound may be of therapeutic importance in autoimmune, inflammatory and neurodegenerative diseases.

## Materials and Methods

### Reagents

Cell culture materials (DMEM/F-12, L-glutamine, Hanks’ balanced salt solution, 0.05% trypsin, and antibiotic/antimycotic) were purchased from Thermo Fisher. Fetal bovine serum (FBS) was obtained from Atlas Biologicals. Benzyl cinnamate (cinnamein), Griess reagent, bacterial lipopolysaccharides (LPS) and polyinosinic-polycytidilic acid (poly IC) were procured from Sigma-Aldrich (St. Louis, MO).

### Isolation of primary mouse microglia and astroglia

Primary glial cells were isolated from mixed glial cultures following the procedure of as described before [9–14]. Briefly, cerebral tissues collected from 2- to 3-d-old mouse pups were homogenized with glass mortar, triturated, passed through mesh, trypsinized, centrifuged, and mixed glial cells plated in DMEM/F-12 containing 10% fetal bovine serum.

For microglia, on day 9, the mixed glial cultures were washed three times with DMEM/F-12 and subjected to a shake at 240 rpm for 2 h at 37°C on a rotary shaker to remove loosely attached microglia. The cell suspensions were placed on uncoated culture plates for 30 min followed by removal of nonadherent cells by washing. Adherent cells were cultured in DMEM/F-12 containing 10% FBS. More than 98% of these cells stained for microglial marker CD11b.

After removal of microglia on day 9, attached cells were cultured in DMEM/F-12 containing 10% FBS. For purifying astroglia, on day 11, cells were shaken at 180 rpm for 18 h to remove any remaining microglia. The adherent cells were washed and seeded onto new plates for further studies. By immunofluorescence assay, these cells homogeneously expressed glial fibrillary acidic protein (GFAP), a marker of astrocytes [15, 16]. These cells were more than 96% pure astrocytes [17].

### RAW 264.7 macrophages

RAW 264.7 cells (purchased from ATCC) were also maintained in DMEM/F-12 containing 10% FBS.

### Assay for NO Synthesis

Synthesis of NO was determined by assay of culture supernatants for nitrite, a stable reaction product of NO with molecular oxygen. Briefly, supernatants were centrifuged to remove cells, and 100 μl of each supernatant was allowed to react with 50 μl of Griess reagent and incubated at room temperature for 15 min [18, 19]. The optical density of the assay samples was measured spectrophotometrically at 570 nm. Fresh culture media served as the blank. Nitrite concentrations were calculated from a standard curve derived from the reaction of sodium nitrite in the assay.

### ELISA

Levels of TNFα, IL-1β and IL-6 were measured in supernatants using ELISA kits from Thermo Fisher following manufacturer’s protocol as described [12, 14, 20].

### Real-time PCR analysis

Total RNA was isolated from RAW264.7 cells using RNAeasy Mini kit (Qiagen, Germantown, MD). To remove any contaminating genomic DNA, total RNA was digested with DNase. Real-time PCR was carried out in the ABI-Prism7700 sequence detection system (Applied Biosystems, Foster City, CA) using SYBR Select master mix as described earlier [21, 22]. The mRNA expression of the targeted genes was normalized to the level of Gapdh mRNA and data was processed by the ABI Sequence Detection System 1.6 software.

### Statistical Analysis

Statistical analyses were performed with one-way ANOVA using Prism 8 (GraphPad Software). Data represented as mean ± SD. A level of p<0.05 was considered statistically significant.

## Results

### Anti-inflammatory effect of cinnamein in RAW 264.7 macrophages

**Figure 1:**
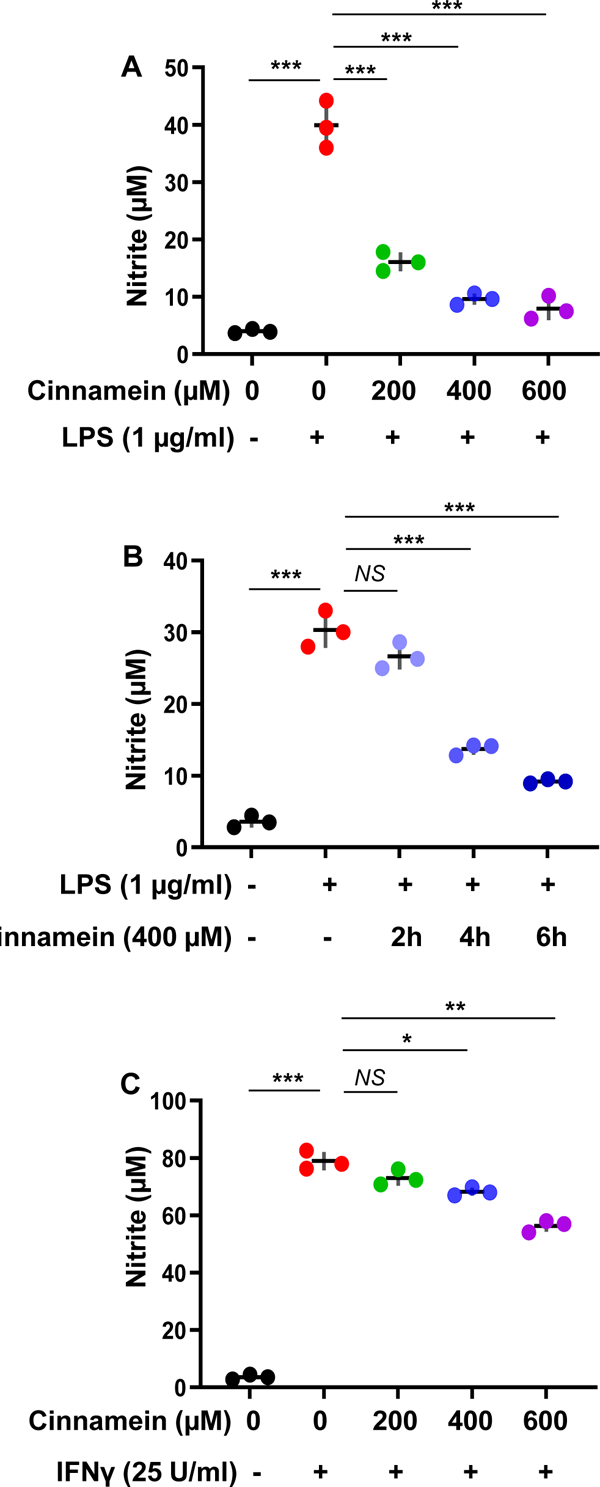
Cinnamein inhibits the induction of NO production from LPS- and IFNγ-stimulated mouse RAW 264.7 macrophages. A) Cells preincubated with different concentrations of cinnamein for 6 h were stimulated with 1 μg/ml LPS under serum-free condition. After 24 h of stimulation, the level of nitrite was measured in supernatants by Griess reagent. B) Cells preincubated with 400 μM cinnamein for different hours were stimulated with 1 μg/ml LPS under serum-free condition. After 24 h of stimulation, the level of nitrite was measured in supernatants. C) Cells preincubated with different concentrations of cinnamein for 6 h were stimulated with 25 U/ml IFNγ under serum-free condition. After 24 h of stimulation, the level of nitrite was measured in supernatants. Results are mean + SD of three independent experiments. *p < 0.05; **p < 0.01; ***p < 0.001; NS, not significant.

Nitric oxide (NO) being released from activated macrophages plays an important role in the pathogenesis of inflammatory disorders [23, 24]. Therefore, to understand the effect of cinnamein on the activation of macrophages, RAW 264.7 cells preincubated with different concentrations of cinnamein for 6 h were challenged with bacterial LPS. As expected, LPS markedly induced the production of NO from RAW 264.7 cells (Fig. 1A). However, cinnamein, at different doses tested, strongly inhibited LPS-induced production of nitrite (Fig. 1A). Next, we examined whether time of incubation had any effect on cinnamein-mediated inhibition of NO in LPS-stimulated RAW 264.7 cells. It was found that 2h preincubation with cinnamein was not sufficient for significant inhibition of LPS-induced NO production (Fig. 1B). However, significant inhibition of NO production was seen when RAW 264.7 cells were preincubated with cinnamein for 4h and 6h (Fig. 1B).

In addition to LPS, many inflammatory stimuli including proinflammatory cytokine interferon γ (IFNγ) are capable of inducing the release of NO from macrophages. Therefore, we examined whether the inhibition of NO production by cinnamein was specific for only LPS or not. As expected, very strong induction of NO production by IFNγ was observed in RAW 264.7 cells (Fig. 1C). Similar to the inhibition of LPS-induced NO production, cinnamein also inhibited IFNγ-induced production of NO in RAW 264.7 cells (Fig. 1C). However, as compared to LPS-induced NO production, cinnamein was less effective in inhibiting NO production from IFNγ-stimulated RAW 264.7 cells (Fig. 1C).

**Figure 2:**
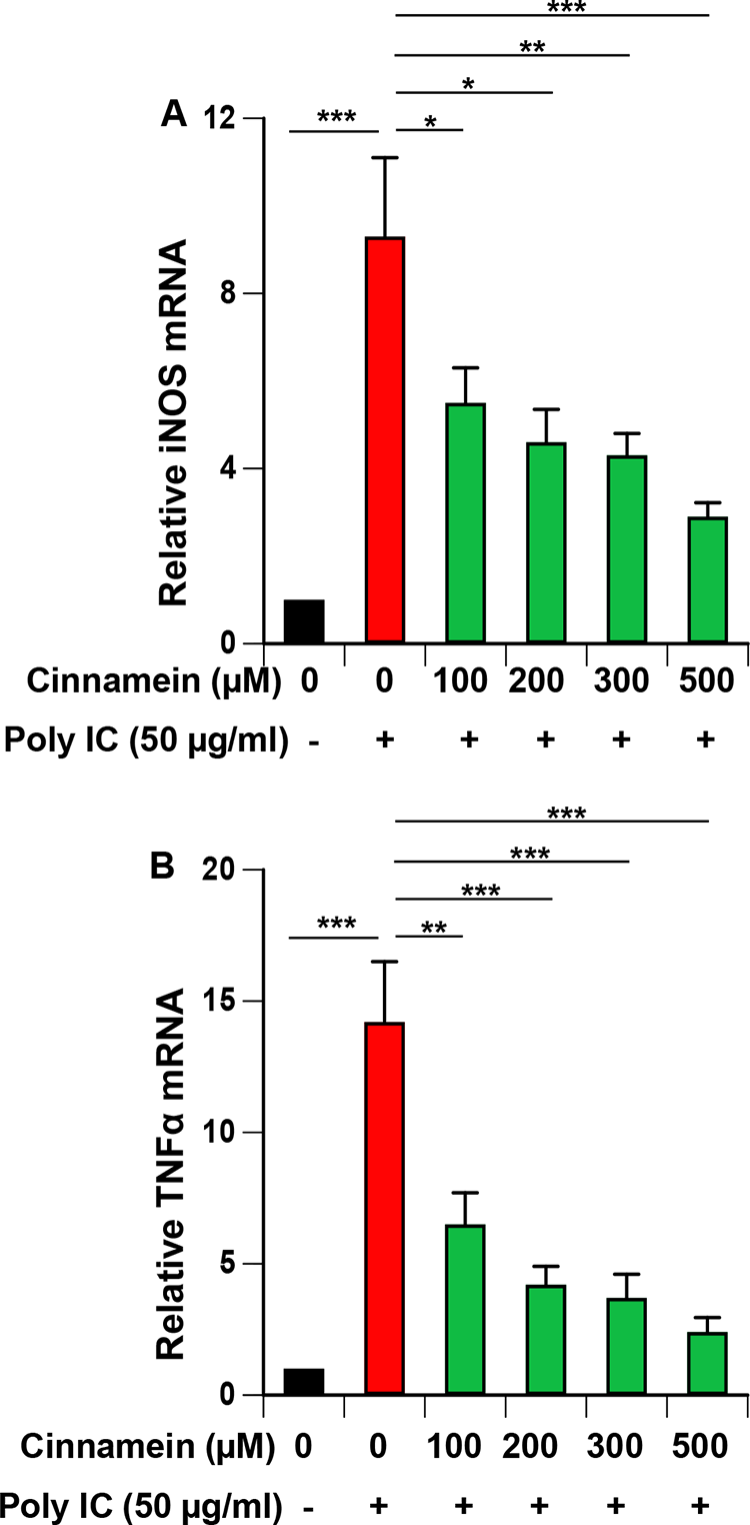
Cinnamein inhibits the mRNA expression of inducible nitric oxide synthase (iNOS) and TNFα in polyIC-stimulated mouse RAW 264.7 macrophages. Cells preincubated with different concentrations of cinnamein for 6 h were stimulated with 100 μg/ml poly IC under serum-free condition. After 12 h of stimulation, the mRNA expression of iNOS (A) and TNFα (B) was monitored by real-time PCR. Results are mean + SD of three independent experiments. *p < 0.05; **p < 0.01; ***p < 0.001.

The enzyme inducible nitric oxide synthase (iNOS) is responsible for the induction of NO production from activated macrophages, microglia, etc. [18, 19]. Therefore, we investigated whether cinnamein could inhibit the mRNA expression of iNOS in RAW 264.7 cells. Poly IC treatment markedly increased the mRNA expression of iNOS in RAW 264.7 cells that was inhibited by different doses of cinnamein (Fig. 2A). Along with iNOS, activated macrophages also express different proinflammatory cytokines including TNFα. Accordingly, poly IC treatment also upregulated the mRNA expression of TNFα in RAW 264.7 cells and similar to the inhibition of iNOS, cinnamein inhibited poly IC-induced TNFα expression (Fig. 2B).

### Cinnamein inhibits the induction of proinflammatory cytokines in primary mouse microglia.

**Figure 3:**
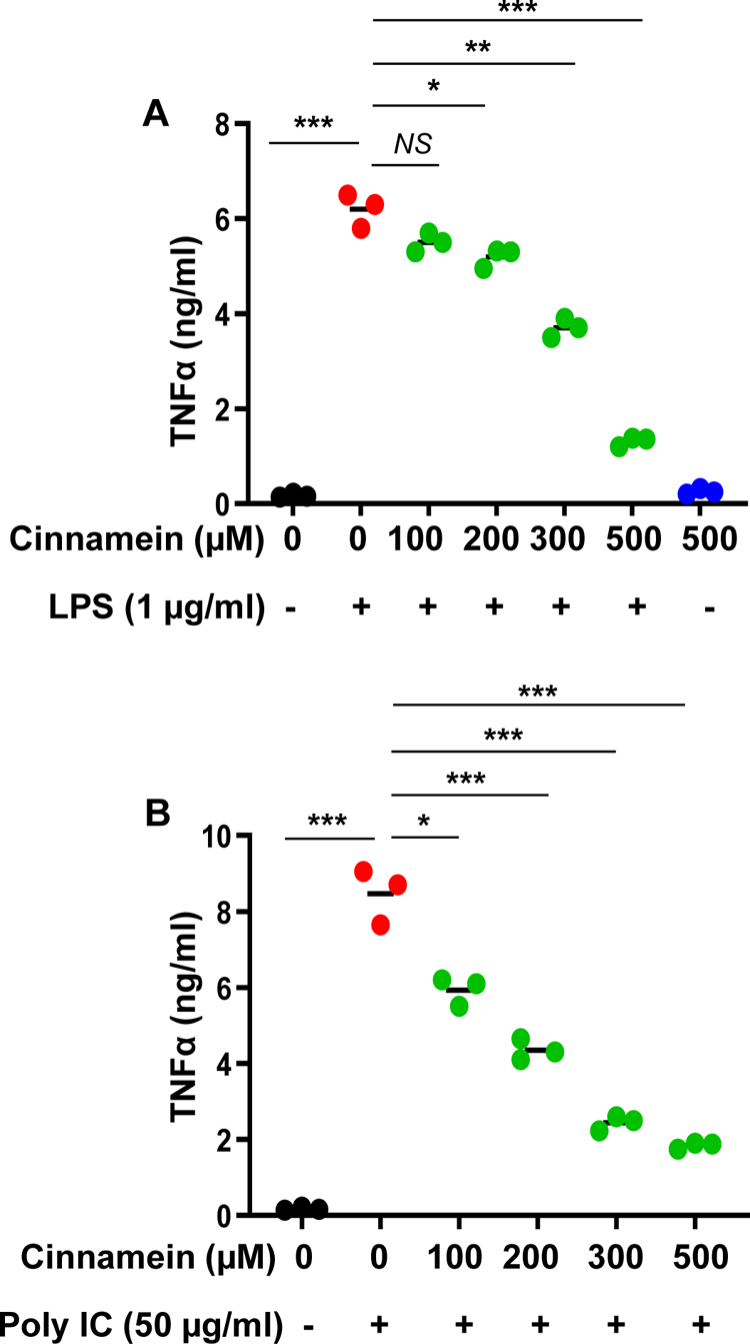
Cinnamein inhibits LPS- and polyIC-induced production of TNFα in primary mouse microglia. Microglia isolated from 2d old mouse pups were incubated with different concentrations of cinnamein for 6 h followed by stimulation with either 1 μg/ml LPS (A) or 50 μg/ml polyIC (B) under serum-free condition. After 24 h of stimulation, the level of TNFα was measured in supernatants by ELISA. Results are mean + SD of three independent experiments. *p < 0.05; **p < 0.01; ***p < 0.001; NS, not significant.

**Figure 4:**
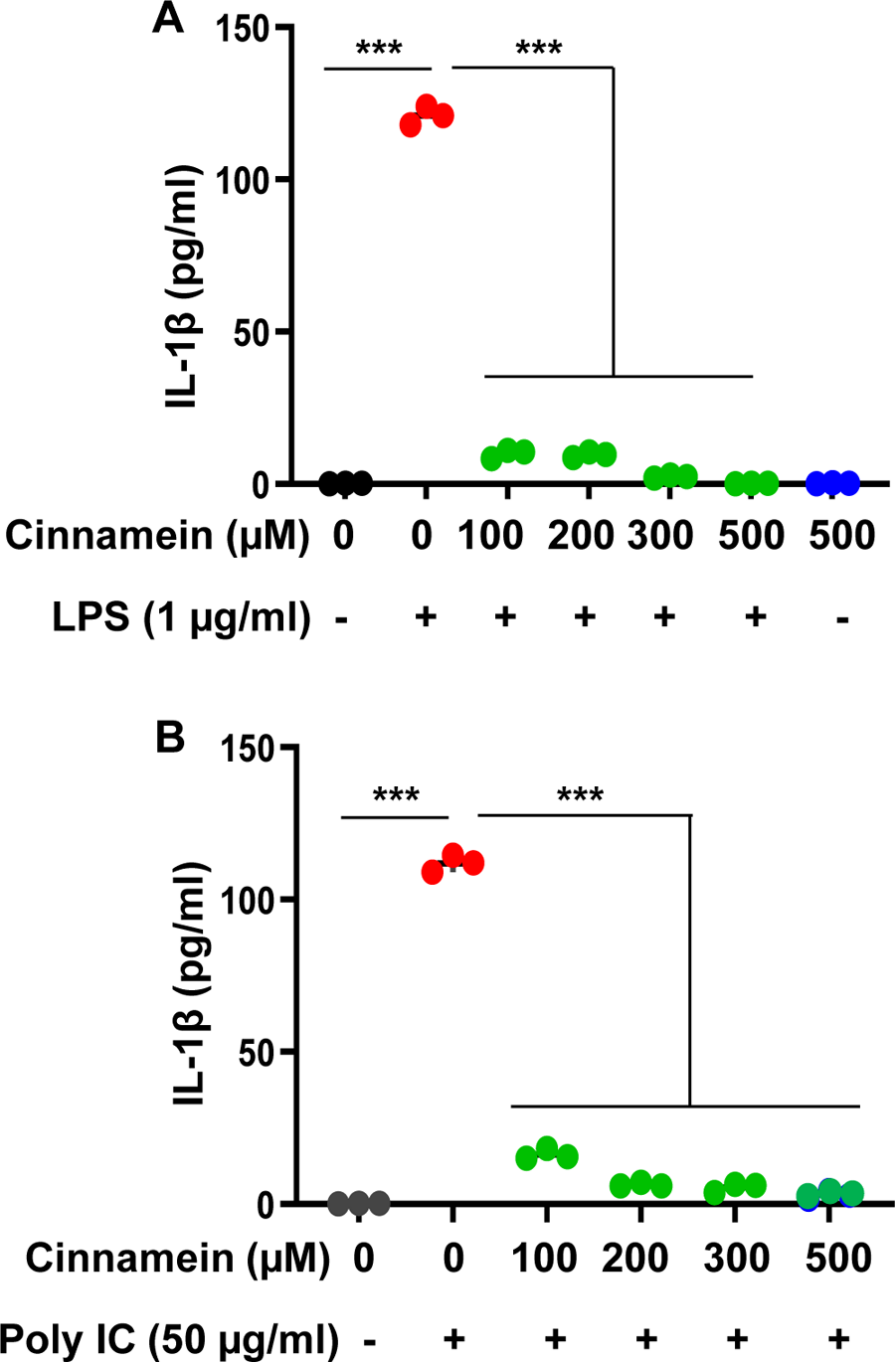
Cinnamein suppresses the production of IL-1β from LPS- and poly IC-stimulated primary mouse microglia. Cells preincubated with different concentrations of cinnamein for 6 h were stimulated with either 1 μg/ml LPS (A) or 50 μg/ml polyIC (B) under serum-free condition. After 24 h of stimulation, the level of IL-1β was measured in supernatants by ELISA. Results are mean + SD of three independent experiments. ***p < 0.001.

**Figure 5:**
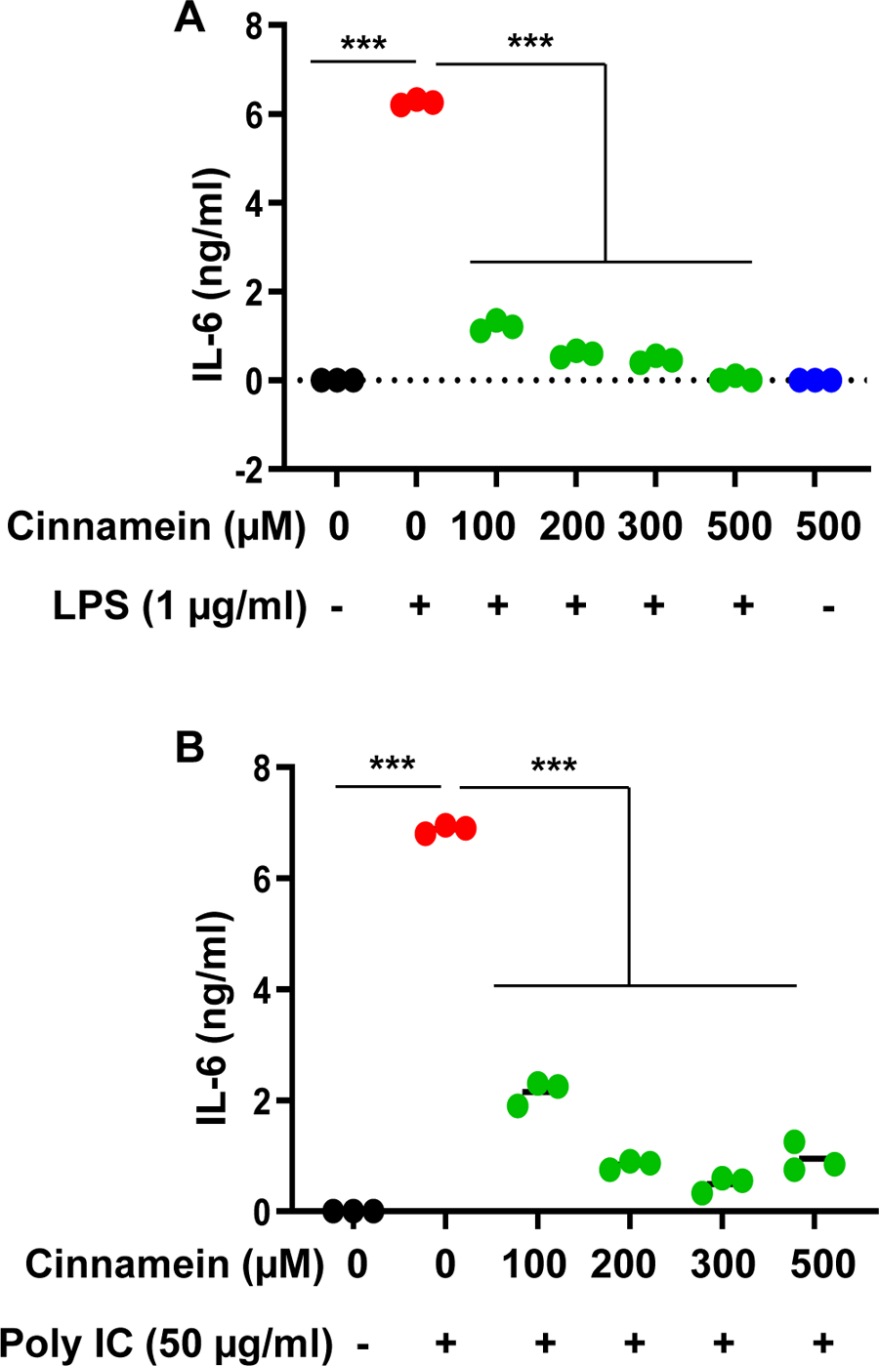
Cinnamein decreases LPS- and polyIC-induced production of IL-6 in primary mouse microglia. Microglia were incubated with different concentrations of cinnamein for 6 h followed by stimulation with either 1 μg/ml LPS (A) or 50 μg/ml polyIC (B) under serum-free condition. After 24 h of stimulation, the level of IL-6 was measured in supernatants by ELISA. Results are mean + SD of three independent experiments. ***p < 0.001.

Microglia are the resident macrophages of the CNS and numerous studies have demonstrated that microglial activation plays an important role in neurodegenerative and neuroinflammatory disorders [25–27]. Therefore, we examined if similar to the suppression of NO and TNFα, cinnamein can also reduce different proinflammatory molecules in mouse primary microglia. Challenge of microglia with LPS led to marked increase in production of TNFα (Fig. 3A), IL-1β (Fig. 4A) and IL-6 (Fig. 5A). Similar to that found with NO production, cinnamein pretreatment attenuated LPS-induced production of TNFα (Fig. 3A), IL-1β (Fig. 4A) and IL-6 (Fig. 5A) in mouse primary microglia. However, as compared to TNFα (Fig. 3A), stronger inhibition was found in case of IL-1β (Fig. 4A) and IL-6 (Fig. 5A). Similar to LPS, viral double-stranded RNA mimic poly IC was also capable of inducing the production of TNFα (Fig. 3B), IL-1β (Fig. 4B) and IL-6 (Fig. 5B) in mouse microglia. In this case as well, cinnamein pretreatment markedly inhibited poly IC-mediated production of TNFα (Fig. 3B), IL-1β (Fig. 4B) and IL-6 (Fig. 5B) in primary microglia.

### Suppression of proinflammatory cytokines in primary mouse astrocytes by cinnamein

**Figure 6:**
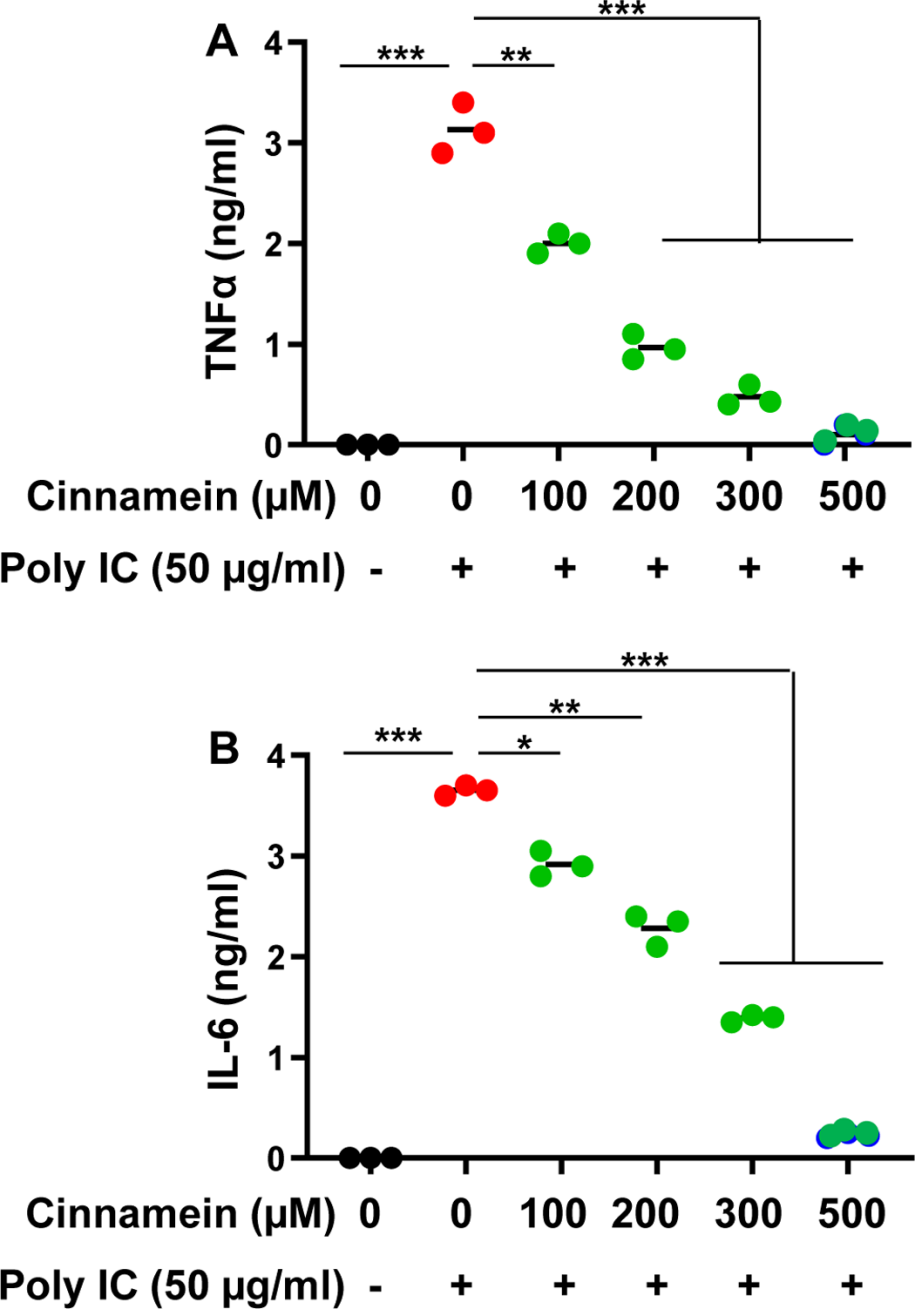
Cinnamein inhibits the production of proinflammatory cytokines from polyIC-stimulated primary mouse astrocytes. Astrocytes preincubated with different concentrations of cinnamein for 6 h were stimulated with 50 μg/ml poly IC under serum-free condition. After 24 h of stimulation, levels of TNFα (A) and IL-6 (B) were measured in supernatants by ELISA. Results are mean + SD of three independent experiments. *p < 0.05; **p < 0.01; ***p < 0.001.

Astrocytes are major cell type in the brain and studies have revealed that these cells also respond to inflammatory challenges to secrete a number of proinflammatory molecules [18, 24, 28, 29]. Therefore, we studied if cinnamein could also decrease the release of different proinflammatory molecules from activated mouse primary astrocytes. Stimulation of astrocytes by poly IC resulted in the production of proinflammatory cytokines like TNFα (Fig. 6A) and IL-6 (Fig. 6B). However, similar to RAW 264.7 macrophages and primary microglia, cinnamein pretreatment significantly inhibited poly IC-mediated production of TNFα (Fig. 6A) and IL-6 (Fig. 6B) in mouse primary astrocytes. Taken together, these results suggest that cinnamein is capable of suppressing proinflammatory molecules in primary glial cells.

## Discussion

Familiar pathological characteristics of various neurodegenerative diseases are massive activation of microglia and astroglia associated with or followed by the loss of invaluable neurons [30–32]. Several studies have established the fact that glial cell-derived proinflammatory response tends to worsen and drive the pathogenic developments leading to neuronal demise. Normally, in the brain, microglial activation has an important repairing function through clearing of unwanted bodies [31, 32]. Similarly, activation of astrocytes may also have important beneficial effects in the retrieval of injured CNS by actively nursing and governing the ion homeostasis, extracellular water, pH, etc. [24, 33]. However, once microglial and astrocytic activation go beyond control in a neurodegenerating microenvironment, detrimental effects of glial activation override its beneficial effects. Activated microglia and astrocyte are known to release proinflammatory cytokines, NO, reactive oxygen species, etc. in excessive amount for a prolonged time period that ultimately damage neurons and oligodendrocytes [34–36]. Hence, controlling microglial and astroglial activation with a nontoxic molecule is an important area of research as that will boost the possibility of finding a therapeutic approach against incurable neurodegenerative disorders.

Being a major component of Balsam of Peru, cinnamein or benzyl cinnamate is used in drinks such as, coffee, flavored tea, wine, beer, juices, etc. for flavoring [37]. Different foods such as chocolate, vanilla, pastries, pudding, ice cream, sauce, etc. finds the use of Balsam of Peru. It is also used in perfumes, colognes, deodorants, soaps, shampoos, conditioners, after-shave lotions, etc. for fragrance. Moreover, different medical products such as cough suppressants, lozenges, ointments, dental cement, etc. also contain Balsam of Peru. Several lines of evidence presented in this study clearly support the conclusion that cinnamein is anti-inflammatory to suppress different proinflammatory molecules in macrophages and brain cells. Our conclusion is based on the following observations. First, LPS, a prototype inducer of inflammation in many cell types including macrophages, induced the expression of iNOS and the production of NO in RAW 264.7 macrophages. However, cinnamein reduced LPS-induced expression of iNOS and generation of NO in macrophages. Second, we extended the study beyond LPS and examined if cinnamein was capable of suppressing inflammation induced by other stimuli and etiological reagents of various neurodegenerative disorders. It is important to find that cinnamein attenuated iNOS/NO in macrophages induced by poly IC (related to viral neuropathy) and IFNγ (related to neuroinflammation and MS). Third, cinnamein also suppressed the production of proinflammatory cytokines (TNFα, IL-1β and IL-6) in mouse primary microglia and astrocytes. Because these proinflammatory molecules have been implicated in the pathogenesis of demyelinating and neurodegenerative diseases, our results provide a potentially important mechanism whereby cinnamein may reduce neural injury [38, 39].

There are several advantages of cinnamein over other proposed anti-neuroinflammatory and anti-neurodegenerative therapies. First, cinnamein is fairly nontoxic. As a constituent of Balsam of Peru and Tolu Balsam, it is being used in foods, drinks, medical products, etc. without any noticeable side effects. On the other hand, commonly-used anti-inflammatory medications such as Tylenol and Ibuprofen cause problems of the liver and stomach, respectively [1–3]. Anti-inflammatory and immunomodulatory medications of MS also exhibit stomach pain, diarrhea, headache, liver injury, lung infection, breathing problems, wheezing, chest pain, urinary tract infection, vaginitis, nausea, vomiting, flu-like symptoms, etc. [40, 41]. Second, oral uptake of a drug is the least painful route and cinnamein can be taken orally. Third, cinnamein could be economical as compared to other existing anti-neurodegenerative therapies.

In summary, we have demonstrated for the first time that cinnamein (a major component of Balsam of Peru and Tolu Balsam) inhibits the production of different proinflammatory molecules from macrophages, microglia and astrocytes. These results highlight an undiscovered property of cinnamein and indicate that this naturally-available compound may find its therapeutic use in neurodegenerative disorders as primary or adjunct therapy.
